# Latin America And Spain: A Successful Partnership

**DOI:** 10.5935/1518-0557.20140001

**Published:** 2014

**Authors:** Miguel Angel Checa

**Affiliations:** Human Reproduction Section Hospital del Mar. IRDC.CIRH. Barcelona Reserach Infertility Group (GRI-BCN). Universitat Autónoma de Barcelona. Barcelona Spain.

Several years ago, our university was invited by REDLARA-ALMER to give a lecture on our polycystic ovary research. During the same year, I met a large number of excellent professionals in Bogotá who were performing work similar to ours as part of a collective effort to “bring happiness to many couples.” Researchers from Europe or the U.S. often consider researchers from Latin America and Spain to possess inadequate academic training. However, after sharing some time with those who I now consider my Latin American friends, I quickly realized that this generalization was not accurate. This characterization speaks more to form, not content. Human reproduction techniques are increasingly standardized, and knowledge is instantly accessed by all through the internet. Before the emergence of new techniques, standards, or treatments, we can access previous publications to ensure comparable results worldwide. I quickly noted that good results are offered by Latin American clinics.

During the previous year, the Autonomous University of Barcelona (UAB) began offering a Masters Program in Human Reproductive Medicine; the curriculum contains a significant amount of clinical scientific content and targeted methodology, research, and medical issues. Hence, the idea of signing an agreement with UAB REDLARA-ALMER emerged in an attempt to provide enhanced methodological training. This collaboration will allow Latin American students to advance their knowledge and clinical practice to the standards demonstrated in international journals. In addition, you can observe good practices occurring in Latin America. The Latin American teachers have visited our university three times to be recognized as teachers to participate in the Masters Program. It’s been three years since the first students arrived from South America, and most of these students are already working across the ocean, with one student is working at a Spanish breeding center.

The results were immediate, as the students began presenting scientific papers in ESHRE (Highly Communicative Presentation by Dr. Roque, who is now a good friend). In addition, the students have published five articles in various scientific journals (Fertility and Sterility, Reproductive Biomedicine Online, American Journal of Assisted Reproduction and Genetics, and Revista Iberoamericana Fertility). Currently, two more articles and reviews from fourth edition students are in preparation. The alliance has been beneficial, as the professionals have been incorporated into their existing or new centers of work. The professionals now possess the knowledge to academically provide what they have done so well over the years (the content). Now, their knowledge and practices can be disseminated worldwide. This collaboration with the UAB Masters Program is currently working with the registry and on a project for new international research.

I can only express our gratitude to those colleagues in Latin American who are now more than friends. We are not only united as Latinos or by language but also as scientists working on a common project.

**Miguel Angel Checa**

Human Reproduction Section

Hospital del Mar. IRDC.CIRH.

Barcelona Reserach Infertility Group (GRI-BCN). Universitat Autónoma de Barcelona. Barcelona Spain.

